# Endocytosis Mediated by *Candida albicans END3* Is Required for Its In Vivo Virulence as an Opportunistic Fungal Pathogen

**DOI:** 10.3390/microorganisms14030598

**Published:** 2026-03-07

**Authors:** Miranda Yu, Cameron Gilmore, Elena Dos Santos, Susan Eszterhas, Samuel A. Lee

**Affiliations:** 1 Medicine Service, White River Junction VA Medical Center, Hartford, VT 05009, USA; miranday@med.unr.edu (M.Y.); cameron.c.gilmore.25@dartmouth.edu (C.G.); elena.b.dos.santos.med@dartmouth.edu (E.D.S.); susan.k.eszterhas@dartmouth.edu (S.E.); 2 School of Arts & Sciences, Dartmouth College, Hanover, NH 03755, USA; 3 Department of Medicine, University of Nevada Reno, Reno, NV 89250, USA; 4 Department of Medicine, Geisel School of Medicine at Dartmouth, Hanover, NH 03755, USA

**Keywords:** *Candida albicans*, *END3*, endocytosis, protein secretion, secreted aspartyl proteases, virulence

## Abstract

Endocytic trafficking in *Candida albicans* is a fundamental cellular process that is crucial for its secretion, filamentation, and virulence-related processes. We have previously demonstrated that loss of the key endocytosis-related *C. albicans* gene *END3* disrupts clathrin-mediated endocytosis, leading to impairments in actin patch formation, filamentation, biofilm formation, cell wall integrity, and extracellular protease secretion. The *end3* null mutant also exhibits altered antifungal susceptibility and reduced host-cell damage in an in vitro keratinocyte infection model. To ascertain whether endocytosis is required for virulence in vivo, we assessed virulence of the *C. albicans end3* null mutant in a murine model of disseminated candidiasis. After infection via the tail vein, and analysis of host survival over 28 days, the *end3* null mutant was markedly hypovirulent compared to corresponding control strains. These results indicate that endocytosis mediated by *END3* in *C. albicans* contributes to pathogenesis in vivo.

## 1. Introduction

The opportunistic fungal pathogen *Candida albicans* is a common cause of hospital-acquired bloodstream infections (BSIs), and is a major cause of catheter-associated infections and invasive disease. Secretory pathways in *C. albicans* enable virulence by conducting secretion of virulence-related proteins, facilitating the highly orchestrated process of polarized secretion during filamentation, and supporting biofilm formation which in turn promotes intravascular infection and dissemination [1,2,3]. However, there is still an incomplete understanding of the molecular mechanisms of trafficking and secretion in *C. albicans*, and of their roles in virulence-related processes and pathogenesis.

In prior work, we demonstrated that pre-vacuolar secretion, mediated by *VPS1*, *VPS4*, and *PEP12*, is required for secretion of Saps and lipases, and also for filamentation and biofilm formation [1,4,5,6]. We also demonstrated that pre-vacuolar secretion is required for virulence through both in vitro and in vivo models of infection. Specifically, we demonstrated that the pre-vacuolar secretory gene *VPS4* is required for virulence in a mouse tail vein model of infection [4]. Similarly, the pre-vacuolar secretory gene *PEP12* is also required for in vivo virulence [5]. We next determined that late secretion plays a key role in *C. albicans* polarized secretion and filamentation; specifically, we determined that the exocyst-related SNARE proteins Sso2p and Sec9p are essential for viability in *C. albicans*, and are required for Sap and lipase secretion and filamentation [7]. Interestingly, many of these anterograde secretory pathways also appear to contribute to retrograde or endocytic pathways [1]. However, despite its importance as a fundamental cellular process, endocytosis has only been studied to a limited extent in *C. albicans*.

Prior studies have shown that the early endocytic coat protein encoded by *EDE1* is non-essential in *C. albicans*. In [8], its null mutant was found to display normal growth and filamentation, though endocytosis was not specifically assayed. We studied the early endocytosis gene *PAL1* and found that mutants lacking *PAL1* were defective in filamentation and biofilm formation, instead forming pseudohyphal cells in filamentation-inducing conditions [9]. Additionally, the *C. albicans* middle coat protein encoded by *SLA2* contributes significantly to filamentous growth [10]. *C. albicans sla2* null mutants are defective in fluid-phase endocytosis and polarized secretion [11]. Lipid rafts normally trafficked to the hyphal tip are mis-localized. *C. albicans sla2* mutants are also defective in Rbt5p-mediated uptake of iron that is based upon endocytosis [12]. Furthermore, they exhibit reduced rates of growth and form enlarged, globular mother cells because of delays in cell cycle progression mediated by the morphogenesis checkpoint kinase Swe1p [13]. In addition, we studied the *C. albicans* ortholog of *ENT1/2* and found that *C. albicans ent2* mutants lacking the middle coat protein encoded by *ENT2* are defective in endocytosis, filamentation, biofilm formation, and tissue invasion in vitro [14].

The key late-coat gene *SLA1* has also been investigated. *C. albicans sla1* mutants were found to exhibit altered filamentation under hyphal-inducing conditions; they produce shorter filaments, along with cortical actin patches that are reduced in number and no longer concentrated at the hyphal tip [15]. Interestingly, in [16], *C. albicans sla1* mutants displayed minimal defects in Lucifer Yellow uptake, but marked defects in FM4-64 internalization to the vacuole and receptor-mediated endocytosis. Another study showed that the *sla1* null mutant strain was significantly attenuated in pathogenicity in a murine infection model [17]. Colonization by the *sla1* mutant was also found to be significantly reduced, compared to colonization by the wild-type strain. Furthermore, the endocytosis gene *PAN1*, which appears to be an essential gene in *C. albicans*, has been studied minimally [8,18]. A conditional *C. albicans MET3p-PAN1/pan1* mutant in repressing conditions developed thick, swollen cells with abnormal filamentation and failure to endocytose FM4-64 [8]. The *C. albicans* homolog of *LAS17*, *WAL1*, has also been studied. In [19], *C. albicans wal1* null mutants were found to be defective in endocytosis, vacuolar morphology, and hyphal development. Although *wal1* mutants were able to conduct polarized morphogenesis, most mutants formed pseudohyphae rather than hyphae.

Our most recent experiments focused on the endocytosis gene *END3*, which encodes a key late-coat endocytosis protein thought to play an important role in Sla1 recruitment and in a Pan1/End3/Sla1 complex [20]. We found that *C. albicans end3* null mutants displayed abnormal actin patch formation, filamentous growth, biofilm formation, cell wall integrity, and extracellular protease secretion [21]. In an in vitro vaginal epithelial model of infection, both *C. albicans ENT2* and *END3* were found to be necessary for tissue invasion, thus revealing a clear link between endocytosis and key virulence-related processes [14,21]. To further define the role of endocytic pathways in pathogenesis, we now seek to define the contribution of the endocytosis gene *END3* to virulence and murine survival in a mouse tail vein model of disseminated candidiasis.

## 2. Materials and Methods

### 2.1. Strains and Media

We previously generated the *end3* mutant and re-integrant strains, and characterized the resultant phenotypes, in earlier work [21]. The parental *C. albicans* strain AHY490 was obtained directly from A. Hernday [22], while the re-integrant strain had been established through homologous recombination of alleles of *END3* into the *end3* null mutant. *C. albicans* strain AHY490, *end3* null mutant (named CREnd3KO), and *END3* re-integrant (named CREnd32KI) were grown at 30 °C in YPD (1% yeast extract, 2% peptone, 2% glucose), or in minimal glucose (0.67% yeast nitrogen base without amino acids [YNB], 2% glucose). Solid media were prepared by adding 2% agar.

### 2.2. Murine Model of Disseminated Candidiasis

All animal experiments were conducted in accordance with institutional guidelines and approved by the White River Junction VA Medical Center Institutional Animal Care and Use Committee (Approval No. 18-A262). BALB/c mice (Charles River Laboratories, Boston, MA, USA) were housed four per sterile rodent microisolator ventilated cage, with wood chip bedding and environmental enrichment (nestlets, plastic huts, and/or tubes). Mice were kept under a 12 h light/dark cycle, humidity levels between 30% and 70%, and temperatures between 68 °F and 79 °F. Mice were also provided with standard mouse chow (Teklad) and plain water *ad lib*. Throughout the study, mice were weighed and monitored twice daily, and were euthanized if they developed any signs of morbidity according to a multi-point scoring system based on clinical observation (Supplemental Figure S1). Mice showing signs of distress such as, but not limited to, weight loss, ruffled fur, excessive huddling, lethargy, shivering and labored breathing were euthanized immediately to minimize pain and suffering, as were mice exhibiting any condition clearly indicating that the animal would not survive. Animals surviving 30 days were also euthanized. All mice were humanely euthanized in an inhalation chamber via inhalation of 100% gaseous CO_2_ from a compressed gas tank introduced at a rate resulting in 30–70% displacement of the air per minute in the chamber. This procedure resulted in rapid induction of unconsciousness (2–3 min) with minimal distress, followed rapidly by cessation of respiration. Mice were maintained under 100% CO_2_ for an additional two minutes prior to cervical dislocation.

In order to assess virulence in a standard mouse tail vein model of invasive candidiasis, the *C. albicans* mutant strains and controls were grown to mid-log phase in YPD at 30 °C. Yeast-phase cells from these strains–parental strain AHY490 from A. Hernday [22], *end3* null mutant (CREnd3KO), and *END3* re-integrant (CREnd3KI) from our lab [21]–were harvested, washed, counted, and re-suspended in sterile 0.9% (*w*/*v*) NaCl. Groups of up to 10 BALB/c male or female mice (Charles River Laboratories, Boston, MA, USA) were injected intravenously with 0.2 mL of each cell suspension at 1.0 × 10^6^ cells per animal, and survival over 28 days was assessed.

We conducted a two-proportion power analysis to estimate the required size of group samples as being 10, achieving 82% power to detect a difference of 0.60000 between the null hypothesis that both group proportions were 0.95000 and the alternative hypothesis that the proportion in group 2 was 0.35000, using a one-sided test with a significance level of 0.05000. We chose to initially over-represent each test condition by using 12 mice in each of the WT-, KO-, and KI-infected groups, in order to account for any failed injections via the lateral tail vein. We confirmed successful intravenous inoculation at time of injection by using established visual and tactile criteria, specifically, visualization of flashback and vein clearing (blanching of the semi-transparent vein during injection), indicating intravascular placement, absence of resistance consistent with intraluminal delivery, and lack of subcutaneous bleb formation. The tail was examined immediately for evidence of extravasation or leakage. Any mouse not successfully inoculated was excluded from the study, resulting in rejection of 3, 2, and 2 mice in each group, respectively.

### 2.3. Statistical Analyses

Kaplan–Meier survival curves were analyzed using the Mantel–Cox test (GraphPad Prism v. 6.0). Data were assessed visually to ensure the test’s assumption of proportional hazards was true. A result was considered statistically significant when *p* < 0.05 compared to other survival curves. Initial weights of mice were analyzed using Student’s *t* test (GraphPad Prism v. 6.0), with significant differences indicated by *p* < 0.01.

## 3. Results

### The end3 Null Mutant Attenuated Virulence in a Murine Model of Infection

**Figure 1:**
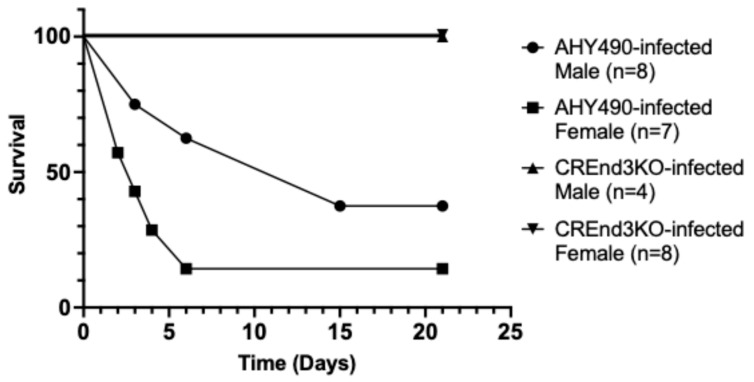
The *C. albicans end3* null mutant (CREnd3KO) is markedly hypovirulent in a mouse model of disseminated candidiasis, compared to a wild-type strain (AHY490). Survival of both male and female mice infected with CREnd3KO was 100%, whereas survival of mice infected with AHY490 was significantly reduced (Mantel–Cox test, *p* < 0.001). Mice were monitored over a period of 21 days post-infusion using a multi-point scoring system as criteria for potential euthanasia. There was no statistically significant difference between the survival rates of male and female mice.

We first assayed the ability of the *end3* null mutant, compared to the wild-type control strain, to infect mice in a tail vein model of disseminated candidiasis as a pilot experiment, comparing male and female mice aged approximately 12 weeks (Figure 1). The mice were infected with either the wild-type strain or the null mutant. The wild-type strain caused significant mortality, with six of seven female mice succumbing to infection over 21 days (median survival 3 days) while five out of the eight male mice did not survive (median survival 15 days). This slight difference in survival between wild-type strain-infected female and male mice–with mortality rates of 85.7% compared to 62.5%—was not statistically significant (Mantel–Cox test, *p* = 0.08). In contrast, all mice infected with the *end3* null mutant survived the infection, as none of the seven females or five males died (Mantel–Cox test, *p* = 0.0002). In this pilot experiment, conducted in a single trial, we did not test the re-integrant strain. The positive control was the parental strain AHY490. A saline-only negative control was not used in the initial pilot study to minimize the number of animals used.

There was a difference in median survival time between male and female mice. However, the initial weights of the male and female mice were also significantly different (Supplemental Figure S2). The male mice were substantially heavier, as determined by a Student’s *t* test (*p* < 0.0001). In contrast, there was no significant difference in starting weights between WT- and KO-infected mice of each sex. The median weights of WT- and KO- infected male mice were 27.8 g and 27.75 g, respectively. For the WT- and KO-infected female mice, the median starting weights were 21.5 g and 21.2 g, respectively.

**Figure 2:**
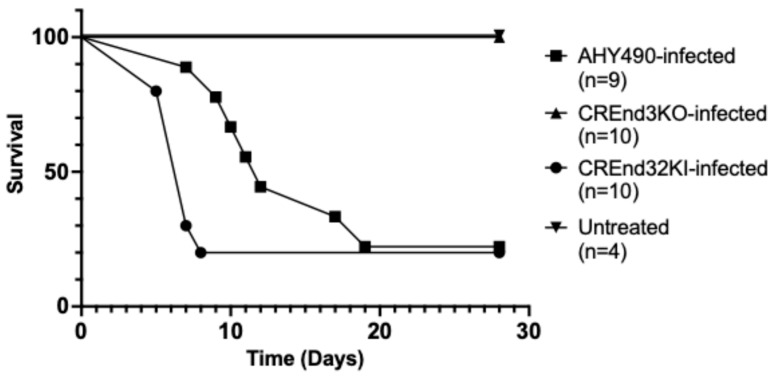
The *C. albicans end3* null mutant (CREnd3KO) is markedly hypovirulent in a female mouse model of disseminated candidiasis compared to the wild-type (AHY490) and re-integrant (CREnd32KI) strains. Survival of female mice infected with CREnd3KO was 100%, whereas survival of female mice infected with the AHY490 and CREnd32KI strains was significantly reduced (Mantel–Cox test, *p* < 0.0001). All the untreated control female mice also survived. Mice were monitored over a period of 28 days post-injection.

Based on these initial results, we performed another experiment in a single trial to compare the *end3* mutant, wild-type (parental), and *END3* re-integrant strains using only female mice (Figure 2) aged approximately 10 weeks. Female mice infected with the wild-type strain, as expected, were vulnerable to infection, with seven of nine mice dying over 28 days (median survival 12 days). The group of 10 mice infected with the *end3* mutant again exhibited no mortality (Mantel–Cox test, *p* < 0.0001). Infection with the re-integrant strain resulted in eight of ten mice dying (median survival 7 days). The slight difference in survival between the wild-type strain and re-integrant strain was not statistically significant (Mantel–Cox test, *p* = 0.92). All four untreated control mice survived.

Additionally, in a post hoc analysis, we combined data from all female mice in both experiments infected with the wild-type strain and compared them to the wild-type-strain-infected male cohort from the first experiment. There was no significant difference in survival outcomes (Mantel–Cox test, *p* = 0.33), a finding which supports our rationale for utilizing a single sex of mice in our main experiment to analyze virulence (Figure 2). In all cases, the difference in survival between the *end3* null mutant and both the wild-type and re-integrant strains was clinically and statistically significant, as none of the *end3* mutant-infected mice died. We observed that both groups of CREND3KO-infected female mice across the two experiments survived completely, along with all CREND3KO-infected male mice in the pilot experiment. These findings demonstrate that the endocytosis gene *END3* is required for wild-type virulence in a tail vein model of disseminated candidiasis.

## 4. Discussion

In prior studies, we demonstrated that the *C. albicans* pre-vacuolar secretory pathway mediated by the vacuolar protein sorting gene *VPS4* is required for secretion of the extracellular proteases Sap2 and Saps4–6 in vitro, and that mutants lacking *VPS4* are dramatically reduced in virulence in a mouse tail vein model of disseminated infection [5]. Interestingly, however, we found that *C. albicans VPS4* is dispensable for virulence in a mouse vaginal model of candidiasis, thus revealing a differential role for the pre-vacuolar secretory pathway in virulence, depending on the infected tissue [23]. We also demonstrated that *PEP12*, which encodes a t-SNARE involved in pre-vacuolar trafficking, is also required for normal biofilm integrity in vitro, and for virulence in a mouse tail vein model in vivo [6].

Because of the intersection of the endocytic pathway with pre-vacuolar and vacuolar trafficking pathways, we subsequently undertook an analysis of the role of endocytosis in virulence-related phenotypes in *C. albicans*. We focused on the key early endocytic genes *ENT2* and *END3*. *ENT2* encodes an epsin-like protein involved in clathrin binding in early endocytosis [24]. In *C. albicans*, we demonstrated that the ent2 null mutant was defective in endocytosis: unable to form proper hyphae and biofilms, and unable to invade vaginal keratinocytes in vitro [14]. It was subsequently demonstrated that a *C. albicans* *tetO*-regulated *ent2* null mutant strain, in the presence of doxycycline, was avirulent in a mouse model of systemic candidiasis (via retro-orbital injection), compared to controls or in the absence of doxycycline [25]. This study did not specifically examine organ fungal burden or histopathology. In this work, we further demonstrate a key role of early endocytosis mediated by *END3* in virulence, using a similar mouse model of disseminated candidiasis (via tail vein injection).

In our initial pilot study, we sought to first determine if there was a difference in survival between female and male mice. We found that there was no statistically significant difference in survival, thus providing reassurance that there were no major biological differences in survival from disseminated infection with *C. albicans* in this model. Of note, the male mice of the same age were larger (by weight) than the female mice, which is likely responsible for the longer median survival of the male mice in this experiment. Moreover, in a large meta-analysis of the clinical literature, sex was not identified as a risk for development of invasive candidiasis in critically ill patients [26]. Taking all these findings together, we believe that use of a single sex of mice is acceptable for conducting virulence studies such as these, and that performance of such experiments using separate male and female mice is unnecessary, particularly in light of the need to limit the overall number of mice used in these experiments in light of ethical considerations.

We found a complete, consistent absence of mortality across all groups of *end3* KO-infected mice, which included both groups of KO-infected female mice, whereas most WT-infected and KI-infected mice did not survive. Collectively, this and preceding studies indicate that early endocytosis in *C. albicans* plays a central role in key virulence-related processes including filamentation and biofilm formation. Additional molecular studies of the role of endocytosis in *C. albicans* pathogenesis are warranted, in order to better understand how this common and dangerous opportunistic pathogen causes morbidity and mortality.

## Data Availability

The original data presented in the study are openly available in Figshare through the following link: https://doi.org/10.6084/m9.figshare.29586365.

## Supplementary Materials

The following supporting information can be downloaded at: https://www.mdpi.com/article/10.3390/microorganisms14030598/s1, Figure S1: The unfilled example of the Daily Mouse Observation Template indicates how the template assesses criteria for euthanasia to minimize pain and suffering in mice; Figure S2: The initial weights in grams of male mice were significantly greater than those of the female mice (Student’s *t* test, *p* < 0.0001).

## Abbreviations

The following abbreviation is used in this manuscript:

Sap	Secreted aspartyl protease

## References

[1] Rollenhagen C., Mamtani S., Ma D., Dixit R., Eszterhas S., Lee S.A. (2020). The Role of Secretory Pathways in *Candida albicans* Pathogenesis. J. Fungi.

[2] Schaller M., Korting H.C., Schäfer W., Bastert J., Chen W., Hube B. (1999). Secreted aspartic proteinase (Sap) activity contributes to tissue damage in a model of human oral candidosis. Mol. Microbiol..

[3] Tsui C., Kong E.F., Jabra-Rizk M.A. (2016). Pathogenesis of *Candida albicans* biofilm. Pathog. Dis..

[4] Lee S.A., Jones J., Hardison S., Kot J., Khalique Z., Bernardo S.M., Lazzell A., Monteagudo C., Lopez-Ribot J. (2009). *Candida albicans* VPS4 is Required for Secretion of Aspartyl Proteases and In Vivo Virulence. Mycopathologia.

[5] Palanisamy S.K., Ramirez M.A., Lorenz M., Lee S.A. (2010). *Candida albicans* PEP12 Is Required for Biofilm Integrity and In Vivo Virulence. Eukaryot. Cell.

[6] Lv Q., Yan L., Jiang Y. (2021). The Importance of Vacuolar Ion Homeostasis and Trafficking in Hyphal Development and Virulence in *Candida albicans*. Front. Microbiol..

[7] Bernardo S.M., Rane H.S., Chavez-Dozal A., Lee S.A. (2014). Secretion and filamentation are mediated by the *Candida albicans* t-SNAREs Sso2p and Sec9p. FEMS Yeast Res..

[8] Martin R., Hellwig D., Schaub Y., Bauer J., Walther A., Wendland J. (2007). Functional analysis of *Candida albicans* genes whose Saccharomyces cerevisiae homologues are involved in endocytosis. Yeast.

[9] Yu M., Ma D., Eszterhas S., Rollenhagen C., Lee S.A. (2023). The Early Endocytosis Gene PAL1 Contributes to Stress Tolerance and Hyphal Formation in *Candida albicans*. J. Fungi.

[10] Asleson C.M., Bensen E.S., Gale C.A., Melms A.S., Kurischko C., Berman J. (2001). *Candida albicans* INT1-induced filamentation in Saccharomyces cerevisiae depends on Sla2p. Mol. Cell. Biol..

[11] Oberholzer U., Nantel A., Berman J., Whiteway M. (2006). Transcript profiles of *Candida albicans* cortical actin patch mutants reflect their cellular defects: Contribution of the Hog1p and Mkc1p signaling pathways. Eukaryot. Cell.

[12] Weissman Z., Shemer R., Conibear E., Kornitzer D. (2008). An endocytic mechanism for haemoglobin-iron acquisition in *Candida albicans*. Mol. Microbiol..

[13] Gale C.A., Leonard M.D., Finley K.R., Christensen L., McClellan M., Abbey D., Kurischko C., Bensen E., Tzafrir I., Kauffman S. (2009). SLA2 mutations cause SWE1-mediated cell cycle phenotypes in *Candida albicans* and Saccharomyces cerevisiae. Microbiology.

[14] Rollenhagen C., Agyeman H., Eszterhas S., Lee S.A. (2021). *Candida albicans* ENT2 Contributes to Efficient Endocytosis, Cell Wall Integrity, Filamentation, and Virulence. mSphere.

[15] Reijnst P., Jorde S., Wendland J. (2010). *Candida albicans* SH3-domain proteins involved in hyphal growth, cytokinesis, and vacuolar morphology. Curr. Genet..

[16] Zeng G., Wang Y.-M., Wang Y. (2012). Cdc28–Cln3 phosphorylation of Sla1 regulates actin patch dynamics in different modes of fungal growth. Mol. Biol. Cell.

[17] Suo C., Gao Y., Yang S., Zhang W., Li C., Ma L., Xu Y., Lei J., Ding C., Li H. (2024). The Endocytosis Adaptor Sla1 Facilitates Drug Susceptibility and Fungal Pathogenesis Through Sla1-Efg1 Regulating System in *Candida albicans*. Infect. Drug Resist..

[18] Wendland B., Emr S.D. (1998). Pan1p, Yeast eps15, Functions as a Multivalent Adaptor That Coordinates Protein–Protein Interactions Essential for Endocytosis. J. Cell Biol..

[19] Walther A., Wendland J. (2004). Polarized Hyphal Growth in *Candida albicans* Requires the Wiskott-Aldrich Syndrome Protein Homolog Wal1p. Eukaryot. Cell.

[20] Sun Y., Leong N., Wong T., Drubin D. (2015). A Pan1/End3/Sla1 complex links Arp2/3-mediated actin assembly to sites of clathrin-mediated endocytosis. Mol. Biol. Cell.

[21] Rollenhagen C., Agyeman H., Eszterhas S., Lee S.A. (2022). *Candida albicans* END3 Mediates Endocytosis and Has Subsequent Roles in Cell Wall Integrity, Morphological Switching, and Tissue Invasion. Microbiol. Spectr..

[22] Nguyen N., Quail M.M.F., Hernday A.D. (2017). An Efficient, Rapid, and Recyclable System for CRISPR-Mediated Genome Editing in *Candida albicans*. mSphere.

[23] Rane H.S., Hardison S., Botelho C., Bernardo S.M., Wormley F., Lee S.A. (2014). *Candida albicans* VPS4 contributes differentially to epithelial and mucosal pathogenesis. Virulence.

[24] Wendland B., Steece K.E., Emr S.D. (1999). Yeast epsins contain an essential N-terminal ENTH domain, bind clathrin and are required for endocytosis. EMBO J..

[25] Lash E., Prudent V., Stogios P.J., Savchenko A., Noble S.M., Robbins N., Cowen L.E. (2023). Ent2 Governs Morphogenesis and Virulence in Part through Regulation of the Cdc42 Signaling Cascade in the Fungal Pathogen *Candida albicans*. mBio.

[26] Thomas-Rüddel D.O., Schlattmann P., Pletz M., Kurzai O., Bloos F. (2022). Risk Factors for Invasive Candida Infection in Critically Ill Patients. Chest.

