# Supplementary figures and images for: Endocytosis Mediated by Candida albicans END3 Is Required for Its In Vivo Virulence as an Opportunistic Fungal Pathogen
Source: Microorganisms. 2026 Mar 7;14(3):598. doi: 10.3390/microorganisms14030598 (PMC13028692; doi:10.3390/microorganisms14030598)

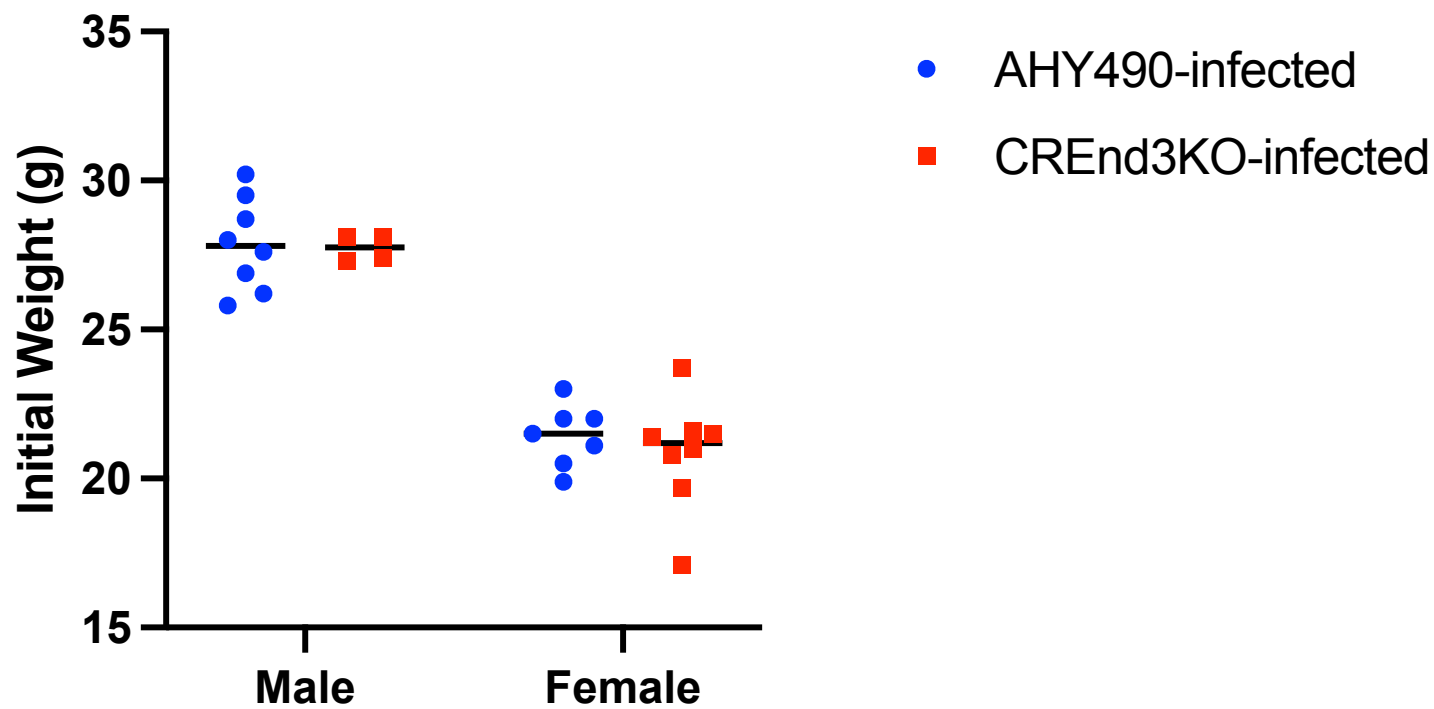

Supplement: Supplementary file 1 [file microorganisms-14-00598-s001.zip › END3 Virulence Supplemental Figure S2.pdf]
